# Role of breast cancer screening in the overdiagnosis of thyroid cancer: results from a cross-sectional nationwide survey

**DOI:** 10.1186/s12905-023-02205-6

**Published:** 2023-02-13

**Authors:** Eunhye Lee, Sung Hoon Jeong, Chung Mo Nam, Jae Kwan Jun, Eun-Cheol Park

**Affiliations:** 1 Department of Radiology, Bucheon Hospital, Soonchunhyang University College of Medicine, 170 Jomaru-Ro, Bucheon, 14584 Republic of Korea; 2 Department of Public Health, Graduate School, Yonsei University, Seoul, 03722 Republic of Korea; 3 Institute of Health Services Research, Yonsei University, Seoul, 03722 Republic of Korea; 4 Department of Preventive Medicine and Institute of Health Services Research, Yonsei University College of Medicine, 50 Yonsei-Ro, Seodaemun-Gu, Seoul, 03722 Republic of Korea; 5 National Cancer Control Institute, National Cancer Center, 323 Ilsan-Ro, Ilsandong-Gu, Goyang, 10408 Republic of Korea; 6 Graduate School of Cancer Science and Policy, National Cancer Center, 323 Ilsan-Ro, Ilsandong-Gu, Goyang, 10408 Republic of Korea

**Keywords:** Asymptomatic, Breast cancer, Mammography, National screening survey, Overdiagnosis, Screening, South Korea, Thyroid cancer, Thyroid cancer screening, Ultrasonography

## Abstract

**Background:**

South Korea has the highest incidence of thyroid cancer worldwide, raising questions regarding the possibility of overdiagnosis. Examining the factors affecting thyroid cancer screening is crucial in elucidating the reasons for this unusually high incidence of thyroid cancer. Therefore, in the present study, we investigated the association between breast cancer screening and thyroid cancer screening to determine the potential role of breast cancer screening in the overdiagnosis of thyroid cancer in South Korea.

**Methods:**

We analyzed the data of women aged > 30 years who were enrolled in the 2014 Korean National Cancer Screening Survey. Self-reported breast cancer screening behavior was categorized as follows: no screening, mammography only, ultrasonography only, and both ultrasonography and mammography. Thyroid cancer screening behavior was categorized as follows: those who had or had not undergone ultrasonography screening. Logistic regression analysis was used to examine the associations between breast and thyroid cancer screening behaviors.

**Results:**

Of the 2270 participants, a total of 569 (25.1%) were screened for thyroid cancer. Those who underwent only mammography, only ultrasonography, or both mammography and ultrasonography were more likely to be screened for thyroid cancer than those who did not undergo breast cancer screening (odds ratio [OR]: 1.47, 95% confidence interval [CI] 1.06–2.04; OR 2.71, 95% CI 1.83–4.02; OR 2.75, 95% CI 1.99–3.80, respectively).

**Conclusions:**

Our findings indicate that thyroid cancer screening in Korea is likely to be performed on an opportunistic basis. Therefore, a nationwide public health and medical initiative is needed to curb the unnecessary use of thyroid screening in the asymptomatic general population.

**Supplementary Information:**

The online version contains supplementary material available at 10.1186/s12905-023-02205-6.

## Background

As shown in the 2018 National Cancer Registration Statistics report, thyroid cancer is the second most common cancer reported in South Korea [1]. Thyroid cancer is three times more common in women than in men and is strongly associated with female hormones [2, 3]. Women often undergo thyroid ultrasonography when being screened for breast cancer, which is the most common cancer among women worldwide [4]. According to the age-standardized cancer incidence rates (2018) in women in Organization for Economic Co-operation and Development (OECD) countries, breast cancer ranked first in all countries except South Korea. Among women in South Korea, thyroid cancer ranked first (62.2 per 100,000 women), followed by breast cancer (57.9 per 100,000 women) [1, 5]. Furthermore, in reports from other Asian countries, thyroid cancer was not ranked among the major cancers, and we note that thyroid cancer was not included among the five major cancers in Japan and China [6]. Hence, the high incidence of thyroid cancer in South Korea is unique and unprecedented. However, there have been no studies or media reports in Korea that radiation exposure, known as a risk factor for thyroid cancer, or iodine intake through seafood has increased. Thus, other factors are likely to involve in the increase in the incidence of thyroid cancer.

Previous studies have indicated that the described increase in thyroid cancer cases could be explained by the increased detection of small thyroid tumors that have no clinical importance, given the extensive use of sensitive screening and accessible diagnostic tools [7, 8]. In addition, despite the high incidence of thyroid cancer, the five-year survival rate of thyroid cancer in South Korea is 100% for both men and women [1]. Therefore, the possibility of overdiagnosis cannot be denied, considering the very high survival rate of thyroid cancer in South Korea, despite its increasing incidence [7, 9, 10]. Furthermore, patients diagnosed with thyroid cancer tend to prefer surgery over being placed under observation for further disease progression, even if the cancer is small in size and does not affect daily function [11]. Therefore, to avoid overdiagnosis of thyroid cancer, data to date indicates that discretionary non-thyroid cancer screening should be avoided if there are no symptoms or risk factors and that thyroid cancer screening using ultrasonography should not be recommended as a routine screening test for asymptomatic adults [7].

To avoid unnecessary screening for thyroid cancer, it is necessary to identify factors that affect initiating thyroid cancer screening. In several previous studies, individual-level factors, including demographic, socioeconomic, and health behavior-related factors, have been examined in regard to their association with thyroid cancer screening; regional-level factors have been examined in a few studies as well [12, 13]. We note that, in South Korea, thyroid cancer screening and breast cancer screening (especially ultrasonography) are often performed together, potentially comprising a reason behind the high diagnosis rate of thyroid cancer. However, the association between the two screening tests in South Korea has not been examined extensively to date. Therefore, the present study aimed to evaluate the association of breast cancer screening with thyroid cancer screening to examine the role of breast cancer screening in the overdiagnosis of thyroid cancer in South Korea.

## Methods

### Data and study population

This study used data collected during the 2014 Korean National Cancer Screening Survey (KNCSS), an annual population-based, cross-sectional survey conducted by the National Cancer Center (from 2004 to the present). This survey aims to determine behavioral patterns related to cancer screening and primarily focuses on stomach, liver, colon, breast, and cervical cancers. The survey also included questions about screening for other cancers, such as lung, prostate, and thyroid cancers [14], and men aged > 40 years and women aged > 30 years who had not been diagnosed with cancer were included in the survey [15]. To ensure that survey participants were representative of the country’s population, the KNCSS used a stratified, multi-step sampling design based on resident populations stratified by geographic area, age, and sex. The sampling method has been described in previous studies [16, 17].

Although the survey has been conducted every year since 2004, the thyroid cancer screening survey has been conducted only three times (in 2009, 2010, and 2014). Since the survey items in 2009 and 2010 were slightly different from those in 2014, only the results of the 2014 survey were used in this study.

**Fig. 1:**
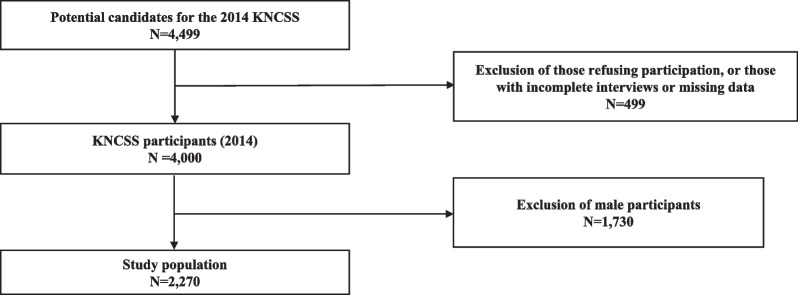
Diagrammatic representation of study participant inclusion. KNCSS, Korean National Cancer Screening Survey

In total, 4499 participants were included in the 2014 survey. The final sample included 2270 women, as 1730 men and 499 participants with incomplete interviews were excluded (Fig. 1). Men were excluded due to the study’s focus on concurrent breast and thyroid cancer screening.

This study was approved by the Institutional Review Board of the National Cancer Center of South Korea (Approval No. NCCNCS-08-129) and was conducted in accordance with the principles of the Declaration of Helsinki and its later amendments. All participants provided written informed consent prior to participation.

### Measures

#### Thyroid cancer screening

The dependent variable in the current study was the presence or absence of thyroid cancer screening. The 2014 KNCSS inquired as to whether the participants underwent thyroid ultrasonography, a common method for thyroid cancer screening. If the answer to the question “Have you ever undergone thyroid ultrasonography in a comprehensive health examination, rectal examination, cancer examination, etc.?” was “yes,” this meant that the respondent had undergone thyroid cancer screening.

#### Breast cancer screening behavior

The independent variable in this study was breast cancer screening behavior. Specifically, the 2014 KNCSS asked questions about breast cancer screening (mammography and ultrasonography). We categorized breast cancer screening behaviors as follows: no screening, mammography only, ultrasonography only, and both mammography and ultrasonography.

### Covariates

In this study, other covariates related to thyroid cancer screening were also included as confounding variables in reference to the results of previous studies [12, 18, 19]. These covariates included age, education level, household income, residential area, occupation, health insurance type, family history of cancer, and participants’ concerns about their risk of cancer. Occupational variables were classified into four categories according to the Korean version of the standard occupational classification system, which is based on the guidelines of the International Labor Organization: white (white-collar), pink (sales and service), blue (agricultural, forestry, fishing, military), and unemployed (housewife, student) [20, 21].

### Statistical analysis

In all analyses, sampling weights were applied to account for complex sampling and to represent the general Korean population with minimal bias [22]. Descriptive statistics were expressed as frequencies (N) and percentages (%), and chi-square tests were performed to evaluate and compare the general characteristics of the study population. After considering potential confounding variables, multiple linear regression analysis was performed to investigate the association between breast cancer screening type and thyroid cancer screening status. Furthermore, a subgroup analysis was performed to determine the relationship between thyroid cancer screening and the type of medical institution that conducted the breast cancer screening. We divided the medical institutions that provided breast cancer screening into the following categories: general hospitals, hospitals, clinics, and screening centers (including organizations that specialize only in screening, such as the Korea Association of Health Promotion and Korea Population and Health Welfare Association; see Additional file 1: Table S1). The results were presented as odds ratios (ORs) and 95% confidence intervals (CIs).

All statistical analyses were performed using the SAS software (version 9.4; SAS Institute, Cary, NC, USA). Statistical significance was set at a two-sided p-value of *p* < 0.05.

## Results

Table 1 shows the general characteristics of the study population by type of breast cancer screening. Of the 2,270 individuals included in the study, 478 (21.1%) did not undergo breast cancer screening, 836 (36.8%) underwent only mammography, 245 (10.8%) underwent only ultrasonography, and 711 (31.3%) underwent both mammography and ultrasonography. In addition, 336 people (14.8%) responded that they had a family history of cancer, whereas 1,934 people (85.2%) responded that they had no family history of cancer. Furthermore, 1,710 (75.3%) respondents answered that they had concerns about their risk of cancer, whereas 560 (24.7%) answered that they did not have concerns.

**Table 1:** General characteristics of the study population

Variables	Thyroid ultrasonography										*P *value
	Total		None		Only mammography		Only ultrasonography		Both		
	N	%	N	%	N	%	N	%	N	%	
	2270	100	478	21.1	836	36.8	245	10.8	711	31.3	
*Age*											< .0001
30–39	559	24.6	235	49.2	147	17.6	64	26.1	113	15.9	
40–49	640	28.2	92	19.2	283	33.9	56	22.9	209	29.4	
50–59	594	26.2	69	14.4	237	28.3	70	28.6	218	30.7	
60–69	350	15.4	57	11.9	122	14.6	41	16.7	130	18.3	
≥ 70	127	5.6	25	5.2	47	5.6	14	5.7	41	5.8	
*Educational level*											0.1865
Middle school or below	338	14.9	73	15.3	131	15.7	33	13.5	101	14.2	
High school	1265	55.7	242	50.6	469	56.1	145	59.2	409	57.5	
College or above	667	29.4	163	34.1	236	28.2	67	27.3	201	28.3	
*Household income*											0.0095
Low	559	24.6	121	25.3	203	24.3	68	27.8	167	23.5	
Mid-low	469	20.7	114	23.8	182	21.8	37	15.1	136	19.1	
Mid-high	671	29.6	148	31.0	247	29.5	62	25.3	214	30.1	
High	571	25.2	95	19.9	204	24.4	78	31.8	194	27.3	
*Residential area*											< .0001
Urban	1037	45.7	243	50.8	409	48.9	76	31.0	309	43.5	
Suburban	1089	48.0	211	44.1	386	46.2	143	58.4	349	49.1	
Rural	144	6.3	24	5.0	41	4.9	26	10.6	53	7.5	
*Occupational*											0.8058
Unemployed	295	13.0	65	13.6	101	12.1	31	12.7	98	13.8	
White	545	24.0	127	26.6	194	23.2	55	22.4	169	23.8	
Pink	176	7.8	38	7.9	65	7.8	16	6.5	57	8.0	
Blue	1254	55.2	248	51.9	476	56.9	143	58.4	387	54.4	
*Health insurance type*											0.1674
NHI	2216	97.6	462	96.7	239	28.6	814	332.2	701	98.6	
MAP	54	2.4	16	3.3	6	0.7	22	9.0	10	1.4	
*Family history*^*a*^											0.4120
Yes	336	14.8	64	13.4	120	14.4	34	13.9	118	16.6	
No	1934	85.2	414	86.6	716	85.6	211	86.1	593	83.4	
*Concern about the risk of cancer*											< .0001
Yes	1710	75.3	307	64.2	653	78.1	192	78.4	558	78.5	
No	560	24.7	171	35.8	183	21.9	53	21.6	153	21.5	

*MAP* Medical Aid Program, *NHI* National Health Insurance

^a^Only includes cancer history of first-degree relatives (parents, brothers, sisters)

Table 2 shows the association between breast cancer screening behavior and thyroid cancer screening after adjusting for potential confounding variables. Compared with participants who had not been screened for breast cancer, we detected a statistically significant association between breast cancer screening and thyroid cancer screening among those who underwent only mammography (OR = 1.47, 95% CI 1.06–2.04), those who underwent only ultrasonography (OR = 2.71, 95% CI: 1.83–4.02), and those who underwent both mammography and ultrasonography (OR = 2.75, 95% CI 1.99–3.80).

**Table 2:** Factors associated with thyroid ultrasonography

Variables	Thyroid ultrasonography						
	Total	Yes		No			
	N	N	%	N	%	OR	95% CI
*Breast cancer screening behavior*							
None	478	60	12.6	418	87.4	**1.00**	
Only mammography	836	185	22.1	651	77.9	1.47	(1.06–2.04)
Only ultrasonography	245	80	32.7	165	67.3	2.71	(1.83–4.02)
Both	711	244	34.3	467	65.7	2.75	(1.99–3.80)
*Age*							
30–39	559	496	88.7	63	11.3	**1.00**	
40–49	640	454	70.9	186	29.1	2.66	(1.91–3.70)
50–59	594	392	66.0	202	34.0	3.16	(2.26–4.40)
60–69	350	256	73.1	94	26.9	2.19	(1.50–3.21)
≥ 70	127	103	81.1	24	18.9	1.45	(0.86–2.43)
*Educational level*							
Middle school or below	338	95	28.1	243	71.9	**1.00**	
High school	1265	310	24.5	955	75.5	0.80	(0.59–1.07)
College or above	667	164	24.6	503	75.4	0.97	(0.69–1.35)
*Household income*							
Low	559	139	24.9	420	75.1	**1.00**	
Mid-low	469	95	20.3	374	79.7	0.81	(0.59–1.11)
Mid-high	671	465	69.3	206	30.7	1.00	(0.75–1.32)
High	571	170	29.8	401	70.2	1.24	(0.94–1.64)
*Residential area*							
Urban	1037	245	23.6	792	76.4	**1.00**	
Suburban	1089	282	25.9	807	74.1	1.06	(0.86–1.31)
Rural	144	42	29.2	102	70.8	1.09	(0.73–1.63)
*Occupational*							
Unemployed	1254	319	25.4	935	74.6	**1.00**	
White	295	64	21.7	231	78.3	0.87	(0.62–1.21)
Pink	545	133	24.4	412	75.6	1.00	(0.78–1.28)
Blue	176	53	30.1	123	69.9	1.23	(0.85–1.78)
*Health insurance type*							
NHI	2216	556	25.1	1660	74.9	**1.00**	
MAP	54	13	24.1	41	75.9	1.20	(0.62–2.31)
*Family history*^*a*^							
Yes	336	119	35.4	217	64.6	1.67	(1.28–2.16)
No	1934	450	23.3	1484	76.7	**1.00**	
*Concern about the risk of cancer*							
Yes	1710	464	27.1	1246	72.9	1.19	(0.92–1.53)
No	560	105	18.8	455	81.3	**1.00**	

*MAP* Medical Aid Program, *NHI* National Health Insurance

^a^Only includes cancer history of first-degree relatives (parents, brothers, sisters)

Table 3 shows the association between thyroid cancer screening and breast cancer screening behavior according to the type of institution performing breast cancer screening, after adjusting for potential confounding variables. The probability of undergoing thyroid cancer screening at a screening center was statistically significantly higher when breast ultrasonography or mammography was performed than when neither was performed (mammography at a screening center: OR = 2.07, 95% CI 1.30–3.31; ultrasonography at a screening center: OR = 2.52, 95% CI 1.53–4.16).

**Table 3:** Association between thyroid cancer screening according to the breast cancer screening institution type and behavior

Variables	Thyroid ultrasonography	
	OR*	95% CI
*Mammogram location*		
None	1.00	
General hospital	1.23	(0.94–1.63)
Hospital	1.28	(0.99–1.64)
Clinics	1.03	(0.66–1.59)
Screening center	2.07	(1.30–3.31)
*Ultrasonography location*		
None	1.00	
General hospital	1.69	(1.25–2.30)
Hospital	2.31	(1.83–2.91)
Clinics	1.57	(0.90–2.74)
Screening center	2.52	(1.53–4.16)

*Adjusted for all covariates

## Discussion

This study investigated the association between breast cancer screening and thyroid cancer screening among 2,270 adult women aged > 30 years who participated in a 2014 cancer screening behavior survey, the KNCSS. The main finding of this study was that those who underwent either mammography, ultrasonography, or both procedures were more likely to be screened for thyroid cancer than those who did not undergo any breast cancer screening. Moreover, at health screening centers, the participants who underwent breast cancer screening were more frequently screened for thyroid cancer than those who did not.

Our main finding has several possible interpretations. The standard method for breast cancer screening is mammography. However, in the case of dense breasts, the accuracy of mammography examination is low; therefore, breast ultrasonography is often performed to compensate for this. Also, when mammography and ultrasound are performed simultaneously, the sensitivity is improved compared with when mammography alone is performed [23]. However, combined screening using mammography and ultrasonography has lower specificity, which increases the rates of false positivity and biopsy [24]. As a result, performing both mammography screening and breast ultrasound screening increases breast cancer detection. Thus, in this population, both tests may have been performed due to health concerns, and this tendency may have affected thyroid cancer screening [25]. The high correlation between breast ultrasonography and thyroid cancer screening may also be due to the anatomical location and characteristics of the ultrasound examination. The breast and thyroid are anatomically located close to each other, and linear probes are commonly used for breast and thyroid ultrasound, making it easy to examine these organs together [26]. In contrast, given the use of a convex probe for abdominal or liver ultrasonography and the fact that the neck is not exposed, it is cumbersome to replace the probe and expose the neck to examine the thyroid gland [27]. Because of this convenience, health care providers and patients are more likely to have combined breast and thyroid cancer screening.

In addition, in our study, women who only received mammograms were more likely to receive ultrasounds for thyroid cancer. In Korea, the National Cancer Screening Program (NCSP), which was implemented in 1999 and screens for gastric, liver, colon, breast, and cervical cancers, is conducted nationwide at no cost or with a small copayment [19]. Although thyroid cancer screening is not covered by the NCSP, health care providers usually offer ultrasound screening for thyroid cancer as an additional low-cost option, ranging from 30 to 50 USD [28]. According to previous studies, women who received cervical and breast cancer screening were more likely to undergo thyroid cancer screening than women who did not participate in cancer screening [18]. Therefore, the health system that promotes cancer screening in Korea may have exerted behavioral effects on the population undergoing mammography [29].

This trend is also expected to be observed frequently in medical institutions that do not have separate specialized fields, such as general hospitals. Accordingly, our subgroup analysis showed that those who underwent mammography or breast ultrasound at screening centers, such as the Korea Health Care Association and the Population Health and Welfare Association, were 2.07 and 2.52 times more likely to be screened for thyroid cancer than those who did not undergo breast cancer screening, respectively. These results objectively illustrate the previously reported challenges of doctor-induced demand and profit-seeking policies of medical care providers in South Korea [7, 28].

Other researchers have similarly questioned whether the main reason behind the marked increase in thyroid cancer cases is excessive thyroid cancer screening [30, 31]. Overdiagnosis starts at the macro level, with mechanisms through which health care services are paid for at the systemic level, comprising an important aspect of overdiagnosis, and extends to the micro level (e.g., the way the findings are examined) [8, 32]. In Korea, after the medical system reform in 2000, thyroid ultrasound has been widely used in local clinics. Furthermore, between 2001 and 2004, the annual number of ultrasound examinations for thyroid cancer doubled, and some hospitals and clinicians recommend regular health examinations, including thyroid cancer screening [33]. Accordingly, the annual cost of thyroid ultrasound examination in Korea is calculated from 121 billion South Korean won (KRW) to 1,490.5 billion KRW [34], which is by no means a small amount. As a result, the possibility of generating profits from the additional costs of thyroid cancer screening, which is not covered by the National Health Insurance, cannot be denied [7].

All cancer screening tests have both benefits and harms. The most important benefit of an effective cancer screening program is a reduction in cancer-specific mortality. In other words, cancer screening is ultimately applied as a measure to reduce the death rate due to cancer. However, it has been confirmed that thyroid cancer screening in Korea only increases the incidence rate of thyroid cancer without affecting the associated mortality rate [35]. The mortality rate of thyroid cancer in Korea is very low, at approximately 0.5 per 100,000 people, and has not changed over the past 10 years [36]. Therefore, the increase in thyroid cancer screening in Korea seems to have been initiated for reasons other than preventing death from cancer. Our finding that participants who had breast cancer screening were more likely to have undergone thyroid cancer screening might be the most plausible explanation for the present state of thyroid cancer overdiagnosis. We also note that the increased incidence of thyroid cancer in South Korea may be due to the detection of mostly small tumors (i.e., tumors < 20 mm in size) [7]. Since most thyroid cancers < 0.5 cm in size have a good prognosis and the treatment benefit is unclear, follow-up is recommended in South Korea [37]. Nevertheless, increased detection of thyroid cancer cases has resulted in more patients undergoing thyroid surgery [11, 35]. Surgery for thyroid cancer is safe for most patients but causes complications such as voice problems or low calcium levels in approximately 1–10% of patients [38–40]. Likewise, these patients must take thyroid hormone replacement drugs for the rest of their lives, which can lead to an increase in national health care costs [35].

To the best of our knowledge, this study is one of the few studies that have evaluated the association between breast cancer screening and thyroid cancer screening using the 2014 KNCSS data, comprising the strength of this investigation in a nationally representative study population [17, 41]. Nevertheless, our findings should be interpreted with caution in the context of the potential limitations of the study. First, errors may have been derived from the survey process or participants’ self-reports, such as regarding immature responses, recording errors, false or non-response errors, recall biases, and other standard errors. However, since the survey was carried out with face-to-face interviews conducted by experienced and professional interviewers, these errors were likely to be minimal [17]. Second, recall bias of self-reported data for cancer screening may have led to an underestimation or an overestimation of the cancer screening rates [42]. However, in several studies, self-reported cancer screening history showed good agreement with medical record data [43, 44]. Third, the association between breast and thyroid cancer screening was investigated using multiple logistic regression analysis. However, since this was a cross-sectional study, the causality of the relationship and the temporal precedence relationship between the variables could not be elucidated. Additional explanatory studies with robust designs are needed to establish the causality of the observed relationship.

## Conclusions

In summary, we found that the rate of breast cancer screening (i.e., mammography alone, ultrasonography alone, or both mammography and ultrasonography) was statistically significantly associated with the rate of thyroid cancer screening in this nationally representative study. Furthermore, the thyroid cancer screening rate was quite high (25.1%), and many of the tests conducted were likely unnecessary. In other words, thyroid cancer screening in South Korea is likely to be performed as an opportunistic-based screening rather than to assess individual risks.

## Supplementary Information

**Additional file 1**. Table S1. General characteristics of screening institutions by breast cancer behavior.

## Data Availability

The dataset supporting the conclusions of this article is available from the corresponding author upon reasonable request.

## Abbreviations

CI: Confidence interval
KNCSS: Korean National Cancer Screening Survey
KRW: South Korean won
NCSP: National Cancer Screening Program
OECD: Organization for Economic Co-operation and Development
OR: Odds ratio
