# Supplementary material for: Role of breast cancer screening in the overdiagnosis of thyroid cancer: results from a cross-sectional nationwide survey
Source: BMC Womens Health. 2023 Feb 13;23:64. doi: 10.1186/s12905-023-02205-6 (PMC9926730; doi:10.1186/s12905-023-02205-6)
Supplement: Supplementary file 1 — Additional file 1. Table S1. General characteristics of screening institutions by breast cancer behavior. [file 12905_2023_2205_MOESM1_ESM.docx]

| \| **STable 1. General characteristics of screening institutions by breast cancer behavior** \| \| \| \| \| \| \| \| \| --- \| --- \| --- \| --- \| --- \| --- \| --- \| --- \| \| **Variables** \| **Thyroid ultrasonography** \| \| \| \| \| \|  \| \| **Total** \| \| **Yes** \| \| **No** \| \| ***P-value*** \| \| **N** \| **%** \| **N** \| **%** \| **N** \| **%** \| \|  \| 2,270 \| 100.0 \| 569 \| 25.1 \| 1,701 \| 74.9 \|  \| \| **Mammography only** \|  \|  \|  \|  \|  \|  \| <.0001 \| \| None \| 723 \| 31.9 \| 140 \| 19.4 \| 583 \| 80.6 \|  \| \| General hospital \| 527 \| 23.2 \| 144 \| 27.3 \| 383 \| 72.7 \|  \| \| Hospital \| 780 \| 34.4 \| 214 \| 27.4 \| 566 \| 72.6 \|  \| \| Clinics \| 145 \| 6.4 \| 34 \| 23.4 \| 111 \| 76.6 \|  \| \| Screening center^*^ \| 95 \| 4.2 \| 37 \| 38.9 \| 58 \| 61.1 \|  \| \| **Ultrasonography only** \|  \|  \|  \|  \|  \|  \| <.0001 \| \| None \| 1,314 \| 57.9 \| 245 \| 18.6 \| 1,069 \| 81.4 \|  \| \| General hospital \| 271 \| 11.9 \| 82 \| 30.3 \| 189 \| 69.7 \|  \| \| Hospital \| 541 \| 23.8 \| 193 \| 35.7 \| 348 \| 64.3 \|  \| \| Clinic \| 68 \| 3.0 \| 20 \| 29.4 \| 48 \| 70.6 \|  \| \| Screening center^*^ \| 76 \| 3.3 \| 29 \| 38.2 \| 47 \| 61.8 \|  \| |
| --- | --- | --- | --- | --- | --- | --- | --- | --- | --- | --- | --- | --- | --- | --- | --- | --- | --- | --- | --- | --- | --- | --- | --- | --- | --- | --- | --- | --- | --- | --- | --- | --- | --- | --- | --- | --- | --- | --- | --- | --- | --- | --- | --- | --- | --- | --- | --- | --- | --- | --- | --- | --- | --- | --- | --- | --- | --- | --- | --- | --- | --- | --- | --- | --- | --- | --- | --- | --- | --- | --- | --- | --- | --- | --- | --- | --- | --- | --- | --- | --- | --- | --- | --- | --- | --- | --- | --- | --- | --- | --- | --- | --- | --- | --- | --- | --- | --- | --- | --- | --- | --- | --- | --- | --- | --- | --- | --- | --- | --- | --- | --- | --- | --- | --- | --- | --- | --- | --- | --- | --- | --- | --- | --- | --- | --- | --- | --- | --- | --- | --- | --- | --- | --- |
| ^*^ Screening centers include organizations that specialize only in screening, such as the Korea Association of Health Promotion and Korea  Population and Health Welfare Association. |
